# Decreased xCT activity in patients associated with *Helicobacter pylori* infection

**DOI:** 10.3389/fphar.2022.1021655

**Published:** 2022-12-05

**Authors:** Ling Wang, Wen-Qun Li, Fen Liu, Yuan-Jian Li, Jie Du

**Affiliations:** ^1^ Department of Pharmacy, Xiangya Hospital, Central South University, Changsha, China; ^2^ Department of Pharmacy, The Second Xiangya Hospital, Central South University, Changsha, China; ^3^ Department of Digestion, The Third Xiangya Hospital of Central South University, Changsha, China; ^4^ National Clinical Research Center for Geriatric Disorders (XIANGYA), Xiangya Hospital, Central South University, Changsha, China

**Keywords:** gastric ulcer, xCT, glutamate, *HP*, micrornas

## Abstract

**Objective:** In animals, *Helicobacter pylori* (*Hp*)-induced gastric injury is accompanied by a decrease in the activity of the cysteine/glutamate transporter (xCT), which regulates extracellular glutamate levels. However, the impact of xCT activity in patients with Hp infection remains unclear. This study aims to investigate variations of xCT activity in the gastric mucosa of patients with *Hp* infection and to provide a clinical basis for identifying targets related to *Hp* infection.

**Methods:** Our study included a total of 67 patients with gastritis, which consisted of 44 *Hp*-negative and 23 *Hp*-positive peptic ulcer cases. The inclusion criteria used to select patients were as follows: gastric histology was determined with a gastroscope, antral biopsies were taken for urease tests, and pathology and culture were performed for analysis of *Hp*-colonization. The clinical characteristics of the patients were obtained, the expressions of microRNAs and xCT protein were detected using immune histochemical analysis, and the concentration of glutamate in their gastric secretion was determined.

**Results:** The findings revealed that xCT expression was significantly lower in *Hp*-positive patients as compared to *Hp-*negative individuals, which was accompanied by a decrease in glutamate concentration in gastric juice. We also discovered a high expression of microRNAs that have been shown to negatively regulate xCT expression, in *Hp*-positive patients.

**Conclusion:** Reduced xCT activity in patients may play an important role in gastric ulcers caused by *Hp* infection. Our findings suggest that the microRNA/xCT pathway could be a potential treatment target for *Hp*-infection-related ulcers.

## 1 Introduction

*Helicobacter pylori* (*Hp*) is one of the most common pathogens causing chronic gastritis and gastric cancer (FitzGerald and Smith, 2021) and is classified as a clear human carcinogen in the 15th carcinogenic report issued by the US Department of Health and Human Services. The rate of *Hp* infection in the global natural population is over 50%. In China, the rate ranges between 40–90%, with an average of 59% (Kamboj et al., 2017). *Hp* usually colonizes in the mucus layer of the gastric epithelium, causing chronic active gastritis. If left untreated, however, long-term infection with *Hp* may even lead to gastric cancer. As a result, early treatment of *Hp*-related gastritis is critical. Antibacterial drugs combined with proton pump inhibitors are currently the main drug strategy for *Hp* infection. However, the widespread use of antimicrobial regimens has the potential to exacerbate resistance problems (de Brito et al., 2019). Future research on pathogenic mechanisms and alternative treatment targets, therefore, requires immediate attention.

The pathophysiological mechanism of *Hp*-induced peptic ulcer is complex and not fully understood. On the one hand, *Hp* is thought to colonize gastric mucosa cells and secrete virulence proteins to induce oxidative stress or inflammatory response, resulting in increased production of various inflammatory factors that can impair gastric mucosa barrier function (Feng et al., 2020; Lu et al., 2020). On the other hand, *Hp* can inhibit the secretion of endogenous active substances (such as nitric oxide, prostaglandin, epidermal growth factor, etc.) that have a protective effect on the stomach (Echizen et al., 2016; AbdelAziz et al., 2021). Recent research has found that glutamate, an important endogenous active substance, can protect against acute gastric injury induced by multiple factors, including cold-restraint stress (Chen et al., 2001), deoxynivalenol (Wu et al., 2014), and non-steroidal anti-inflammatory drugs (NSAIDs) (Du et al., 2014). However, its potential role in long-term chronic infection remains unknown. Our previous animal studies revealed that *Hp*-induced gastric injury was associated with reduced activity of xCT, a regulator of extracellular glutamate levels (Du et al., 2014; Du et al., 2020). However, the xCT activity in *Hp*-infection patients has not been elaborated. In this study, we explored xCT activity and its upstream regulation by microRNA in *Hp*-infected patients. Through our findings, we intended to provide a clinical basis for identifying *Hp* infection-associated targets.

## 2 Materials and methods

### 2.1 Ethics approval and informed consent statement

The Clinical Research Project was approved by the Institutional Review Board (IRB) of Third Xiangya Hospital, Central South University, Hunan, China (approval number 2015-S109). Because the patients were spread across the country over relatively long distances, we recorded their responses after oral informed consent over the phone.

### 2.2 Patient selection

This study included a total of 67 patients (44 *Hp*-negative and 23 *Hp*-positive gastric ulcer cases) who were diagnosed with gastritis during an endoscopic examination at the Third Xiangya Hospital between December 2015 and February 2016. Patients were enrolled in the study only if they met the following criteria: 1) belonged to the age group 18–75 years; 2) had not taken any NSAIDs or antibiotics 2 weeks before the study; 3) had taken a C13 isotope respiration test; and 4) had their gastric histology, urease and *Hp*-colonization determined. The following criteria were used to exclude patients: 1) a history of gastric or duodenal surgery; 2) active cancer, any acute medical or terminal illness. 3) long-term adverse life history, such as chronic alcohol or tobacco use; and 4) missing clinical indicators. The study protocol was approved by the Ethics Committee of our institution. All patients gave informed consent over the phone before participating in the study. Clinical data on patient characteristics, such as gender, age, position of the ulcer, pathologic grade, and *Hp* detection results were obtained.

### 2.3 Endoscopic examination and *Helicobacter pylor* infection detection

The patients took the 13C-urea breath test before an endoscopic examination. Three specimens were collected during endoscopy from the ulcer margin to perform *Hp*-colonization analysis, rapid urease test, and histological examination respectively. This was followed by the collection of gastric juice (10 ml) under gastroscopy. A positive *Hp* infection was confirmed from at least two of three diagnostic tests, namely rapid urease, 13C-urea breath test, or Giemsa stain. A negative result in all three tests defined the absence of infection.

### 2.4 Histology

**FIGURE 1:**
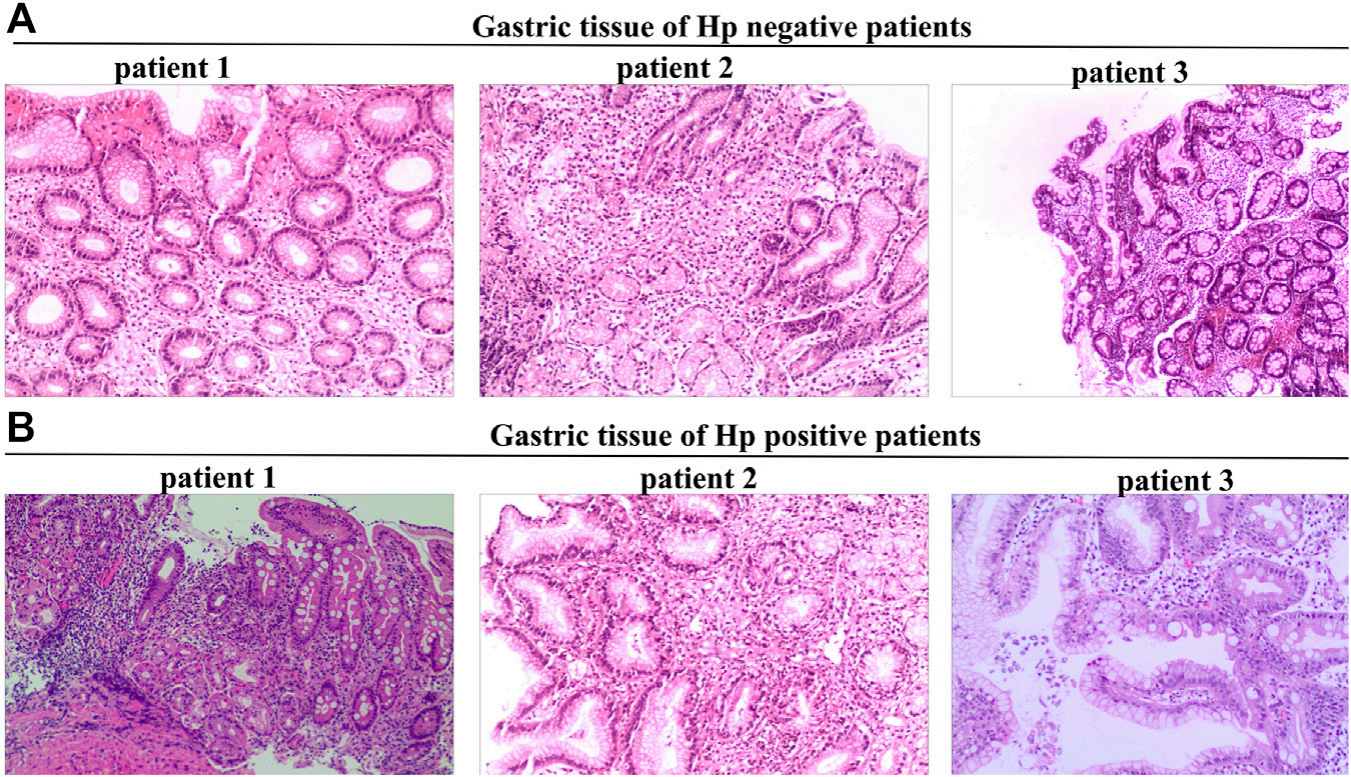
Tissue sections stained with hematoxylin-eosin were used to assess activity, inflammation, atrophy, and intestinal metaplasia. **(A)**, Gastric tissue of Hp negative patients. **(B)**, Gastric tissue of Hp positive patients.

Tissue sections were stained with hematoxylin and eosin to assess activity, inflammation, atrophy, and intestinal metaplasia (Figure 1), and graded on a scale of 0, 1, 2, and 3, corresponding to none, mild, moderate, and severe, respectively, accordingly to the updated Sydney system (Toyoshima et al., 2022). *Hp* colonization in the stomach was measured using Giemsa staining.

### 2.5 Immunohistochemistry and *in situ* hybridization

Tissue sections were used to detect the xCT level by immunohistochemistry (IHC), while miRNA expression was determined using *in situ* hybridization. Acetate buffer (pH 6.0) was used as the immersion solution for the antigen pre-treatment step in IHC staining, while rabbit polyclonal antibody and the Envision (DAKO) polymer detection system were used for staining. Tissue sections were incubated overnight at 4°C with xCT (1:200) antibody, followed by 1-h incubation with anti-rabbit secondary antibody (1:200). *In situ* hybridization was performed using the miRCURY LNA miRNA ISH Kit (Exiqon) and images were acquired using a microscope (Olympus) with a ×20 objective lens.

### 2.6 Determination of glutamate concentration by high-performance liquid chromatography

First, 10 ml of gastric juice was centrifuged at 12000r/min for 3 min and the supernatant was collected. Next, to determine the glutamate content of gastric juice by High-Performance Liquid Chromatography (HPLC), a 10 μL sample or biological reference standard was mixed into 100 μL derivative reagent (a mixture of phthalaldehyde, boric acid buffer, and β-mercaptoethanol). The solution was allowed to react for 3 min at room temperature, transferred to a 2 ml autosampler vial, and analyzed by HPLC. The mobile phase consisted of 0.05 mol/L sodium acetate (pH 6.8), methanol, and, tetrahydrofuran (82:17:1, V/V/V). The flow rate of the column was 1 ml/min at room temperature. The excitation and absorption wavelengths were 338 nm and 430 nm, respectively.

### 2.7 Statistical analysis

All data were expressed as mean ± standard deviations (mean ± SD). The statistical data from all cases were organized using Microsoft Excel. For statistical analysis, SPSS21.0 was used. The counting data were expressed by frequency (n%) and *χ* ^*2*^test. The *t*-test was used to analyze all factors, and *p* < 0.05 was considered statistically significant.

## 3 Results

### 3.1 Clinical characteristics of the patients enrolled in the study

This study included 44 *Hp*-negative and 23 *Hp*-positive peptic ulcer cases. The 44 *Hp*-negative peptic ulcer patients included 22 males and 22 females, with an average age of 51.8 years (51.80 ± 11.1). The 23 *Hp*-negative peptic ulcer patients included 14 males and nine females, with an average age of 45.6 years (45.60 ± 10.7). There was no difference in complications between the two groups. In the *Hp*-positive group, 56.5% of patients had antral ulcers (*n* = 13), 34.8% had corpus ulcers (*n* = 8) and 8.7% had multiple site ulcers (*n* = 2). In the *Hp*-negative group, 61.4% of patients had antral ulcers (*n* = 27), 25.0% had corpus ulcers (*n* = 11) and 13.6% had multiple site ulcers (*n* = 6) (Table 1).

**TABLE 1:** Clinical characteristics of the patients enrolled in the study *n* (%).

Characteristics	*Hp* positive group (*n* = 23)	*Hp* negative group (*n* = 44)	*χ* ^*2*^/*t*	*p*-value
SEX				
Male	14(60.9%)	22(50.0%)	0.718	0.397
Female	9(39.1%)	22(50.0%)		
Age, yr (mean ± SD)	45.60 ± 10.7	51.80 ± 11.1	−2.194	0.032
Position of ulcer				
Corpus	8(34.8%)	11(25.0%)	0.869	0.701
Antrum	13(56.5%)	27(61.4%)		
Fundus	2(8.7%)	6(13.6%)		
Pathologic grade (mean ± SD)				
Inflammation	1.74 ± 0.689	2.00 ± 0.747	−1.393	0.168
Activity	1.70 ± 0.765	1.66 ± 0.645	0.207	0.837
Atrophy	0.22 ± 0.422	0.34 ± 0.479	−1.085	0.283
Metaplasia	/	/	/	/

*Hp: Helicobacter pylori*.

There was no significant difference in gender, site of ulcer, or grade of pathologic score between the *Hp*-negative and *Hp*-positive patients (Table 1), except in age (*χ* ^*2*^/*t* = 2.194, *p* < 0.05). No cases of intestinal metaplasia were recorded.

### 3.2 Decreased xCT activity in *Helicobacter pylor*-positive patients

**FIGURE 2:**
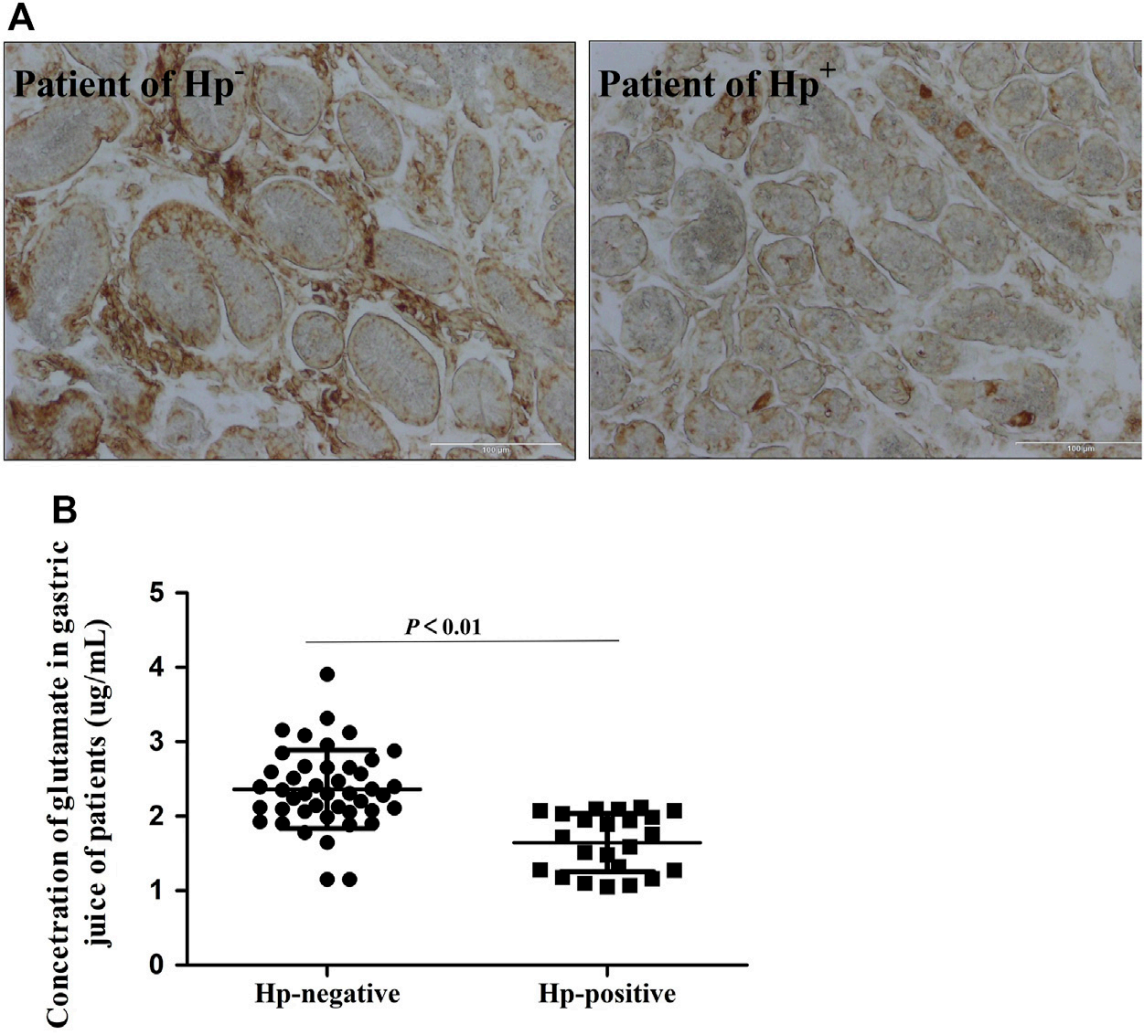
Decreased xCT activity in Hp infection patients. **(A)**. Immunohistochemistry staining for xCT antigen in gastro of *Hp* infection patients. **(B)**. The concentration of glutamate in gastric secretion by HPLC. Data are means ± standard deviations. N = 23–44. *Hp* is short for *Helicobacter pylori*.

Results of IHC staining showed that the level of xCT protein was significantly decreased in *Hp*-positive patients (Figure 2A). We also detected the concentration of glutamate in gastric secretion, which reflects the activity of xCT. Also, the concentration of glutamate in the *Hp*-positive group was significantly lower as compared to the *Hp-*negative group (Figure 2B), which indicates differences in xCT activities between these patient groups.

### 3.3 Higher expression of miR-30b and miR-27a in *Helicobacter pylor-*positive patients

**FIGURE 3:**
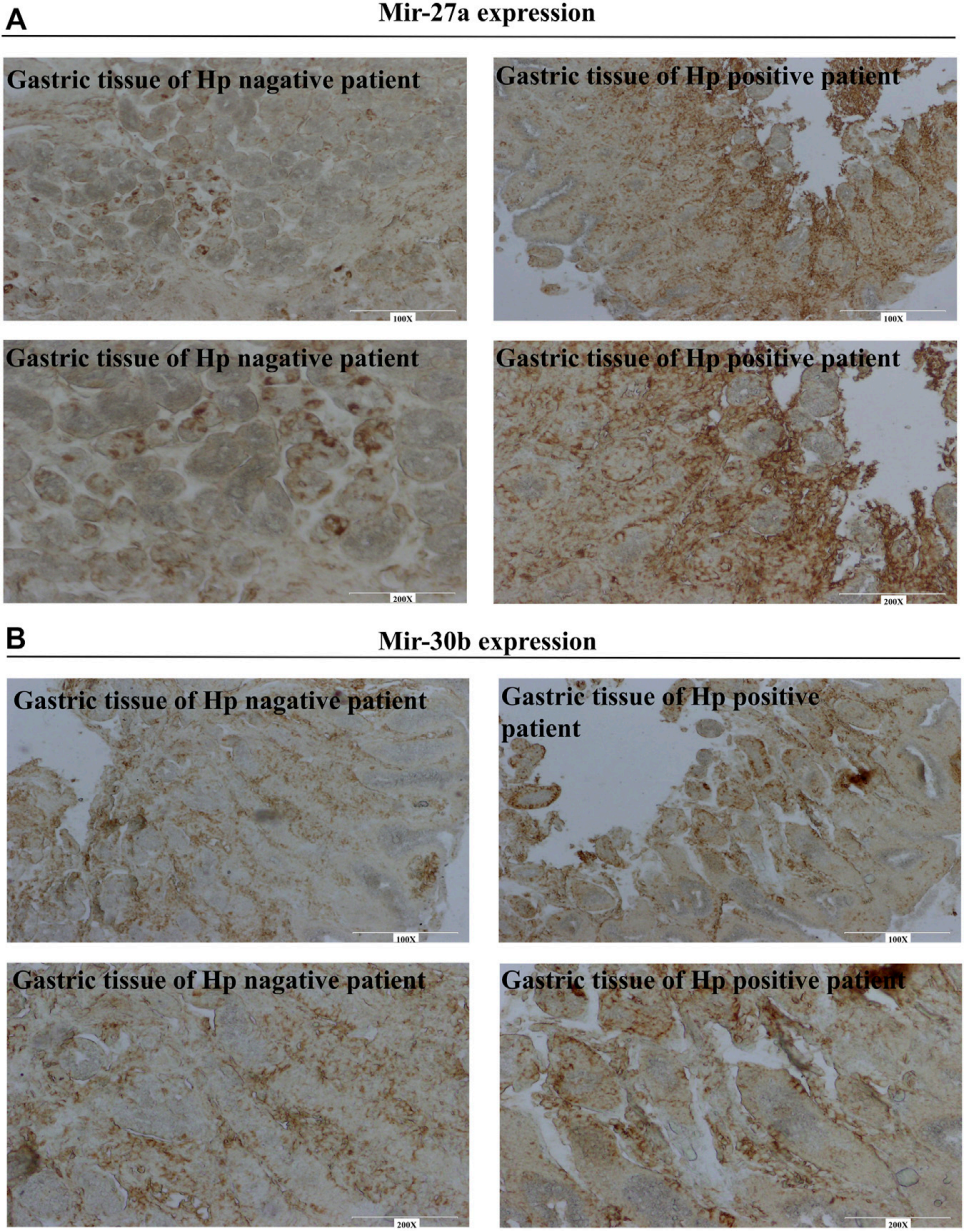
Identifying of miR-27a and miR-30b in gastro of patients using *in situ* hybridization. **(A)** MiR-27a expression in gastro of Hp negative and positive patients. **(B)** MiR-30b expression in gastro of Hp negative and positive patients.

The microRNAs MiR-30b and miR-27a have been reported to downregulate xCT expression. However, the variations of these two miRNAs in patients with gastritis are not known. Figure 3A shows a lower expression of miRNA-27a in *Hp*-negative patients but a higher expression in *Hp*-positive patients. Similar expression patterns were observed for miR-30b (Figure 3B). These results suggested that variation of miRNA/xCT expression may contribute to *Hp* infection in clinical settings.

### 3.4 Correlation between glutamate concentration and pathological grades

For *Hp*-positive patients, ulcers were classified according to their anatomical location, where 56.5% of cases were antral ulcers (*n* = 13), 34.8% of cases were corpus ulcers (*n* = 8), and 8.7% cases were multiple site ulcers (*n* = 2). There was no significant difference between the antrum and corpus of the ulcers. We also analyzed the correlation of glutamate concentration with pathological grades. Moreover, no correlation was observed between glutamate level and pathological grade (Table 2).

**TABLE 2:** Correlation of glutamate concentration with pathologic grade.

Variable		Inflammation	Activity	Atrophy	Position of ulcer
Glutamate concentration	*r*	−0.099	−0.066	0.055	0.080
	*p*	0.654	0.766	0.802	0.717

## 4 Discussion

Glutamate is a non-essential amino acid with significant physiological functions. In the past decades, researchers have discovered a critical role of glutamate in the central nervous system, where it has been recognized as one of the most ubiquitous excitatory neurotransmitters (Platt, 2007; Moroz et al., 2021). Recent studies have also shown evidence of glutamate expression in peripheral organs (Rose, 2002; Valdivielso et al., 2020; Zhang et al., 2021), especially in the stomach (Iijima et al., 2008). It participates in gastrointestinal functions, including regulating oxidative reactions, immune responses, and, barrier function. Abnormal glutamate signaling may lead to gastrointestinal diseases such as gastritis or ulcers. The bacteria *Helicobacter pyroli* and NSAIDs are recognized as the two major causes of gastritis or ulcers. It has been found that decreased glutamate is directly associated with gastric damage in these two gastric ulcer models (Du et al., 2014; Du et al., 2020). Several animal studies have concentrated on the role of xCT/glutamate in gastric ulcers. For example, in cold-restraint animals, glutamate acts on the ionic receptor NMDA to inhibit gastric acid secretion and protects mucous cells (Chen et al., 2001). In our recent study, we also found that reduced xCT activity was involved in an aspirin-induced acute gastric injury, while exogenously supplied glutamate exhibited protective effects on aspirin-induced gastric mucosa injury. The glutamate pathway also protected the gastrointestinal mucosa against *Hp* infection. However, the majority of these findings are based on animal studies, with no human clinical data available. In this study, we found that the expression of xCT in *Hp*-positive patients was significantly lower than that in negative individuals and that variations in xCT expression were accompanied by a reduction of glutamate concentration in gastric juice. Furthermore, we discovered an increase in the expression of miRNAs known to negatively regulate xCT (Drayton et al., 2014; Du et al., 2020). These findings suggested that the miRNA/xCT pathway could be a potential treatment target for *Hp* infection-related ulcers.

Inflammation is one of the most important mechanisms of *Hp*-associated gastritis. On the one hand, during an infection, immune cells are recruited to the infection site to initiate an inflammatory response. On the other hand, the secretion of virulence proteins directly activates the NF-κB pathway and stimulates the secretion of inflammatory factors (Lahner et al., 2018; Yang et al., 2021). Clinical studies have demonstrated higher expression of IL-1β in patients with *Hp* infection (Koosirirat et al., 2010). Animal studies have also shown that multiple inflammatory factors mediate a *Hp* infection, with IL-1β and IL-17 over secretion being the most obvious (Semper et al., 2014)^.^ Some inflammatory factors (such as IL-1β, TNF-α, etc.) have also been proven to affect glutamate transport to other tissues (such as nerve cells, macrophages, etc.) (Ye et al., 2013). For example, IL-1β significantly reduces the expression of glutamate transporter EAAT2 in astrocytes and also inhibits EAAT1 expression in Purkinje cells (Mandolesi et al., 2013). Previous studies have also demonstrated that inflammatory responses can inhibit the activity of xCT (Du et al., 2014; Wu et al., 2014; Shi et al., 2016). Based on these studies, we wanted to look into the causes behind decreased xCT activity for inflammatory regulation in *Hp*-infection patients.

MicroRNAs are single-stranded small RNA molecules that are 19–25 nucleotides in length and silence the expression of target genes through complete or incomplete pairing with the 3′-UTR of the target gene. On being stimulated by an external stimulus, such as a microbial infection, the expression of some miRNAs rapidly changes in the host, affecting the expression of target proteins and regulating the inflammatory immune response process. For example, in *Hp*-induced gastric injury, the expression of mir-155/146b was upregulated, weakening the bacterial clearance from the host (Cheng et al., 2015). *Hp* contributes to chronic inflammation by increasing the expression of miR-328, which targets CD44. (Ishimoto et al., 2015). It has been reported that miRNAs also regulate the expression of xCT. MiR-27a, for example, negatively regulates xCT, thereby mediating cisplatin resistance in patients (Drayton et al., 2014). In a *Hp*-infection animals, miR-30b/27a has been proven to negatively regulate xCT gene expression in luciferase reporter assays (Du et al., 2020). Our clinical findings showed that the expression of miR-27a and miR-30b was significantly increased in *Hp*-positive patients, which is consistent with previous animal studies. Both the literature and our results suggested that the upregulation of miR-30b/27a is involved in the process of *Hp* infection. However, the mechanism by which *Hp* causes these miRNA alterations remains unclear and warrants future studies.

In conclusion, we found that patients with *Hp* infection exhibit reduced expression of xCT and glutamate release, which may play an important role in gastric ulcer induction by the bacteria. Our findings support previous animal studies and suggest that the miRNA/xCT pathway could be a potential treatment target for *Hp* infection-related ulcers.

## Data Availability

The original contributions presented in the study are included in the article/supplementary materials, further inquiries can be directed to the corresponding author.
